# Anti-Metastatic Activity of an Anti-EGFR Monoclonal Antibody against Metastatic Colorectal Cancer with *KRAS* p.G13D Mutation

**DOI:** 10.3390/ijms21176037

**Published:** 2020-08-21

**Authors:** Tomokazu Ohishi, Yukinari Kato, Mika K. Kaneko, Shun-ichi Ohba, Hiroyuki Inoue, Akiko Harakawa, Manabu Kawada

**Affiliations:** 1 Institute of Microbial Chemistry (BIKAKEN), Numazu, Microbial Chemistry Research Foundation and 18-24 Miyamoto, Numazu-shi, Shizuoka 410-0301, Japan; ohbas@bikaken.or.jp (S.-i.O.); inoueh@bikaken.or.jp (H.I.); harakawaa@bikaken.or.jp (A.H.); kawadam@bikaken.or.jp (M.K.); 2 Department of Antibody Drug Development, Tohoku University Graduate School of Medicine, 2-1 Seiryo-machi, Aoba-ku, Sendai, Miyagi 980-8575, Japan; yukinarikato@med.tohoku.ac.jp (Y.K.); k.mika@med.tohoku.ac.jp (M.K.K.); 3 New Industry Creation Hatchery Center, Tohoku University, 2-1, Seiryo-machi, Aoba-ku, Sendai, Miyagi 980-8575, Japan; 4 Institute of Microbial Chemistry (BIKAKEN), Laboratory of Oncology, Microbial Chemistry Research Foundation, 3-14-23 Kamiosaki, Shinagawa-ku, Tokyo 141-0021, Japan

**Keywords:** colorectal cancer, metastasis, epidermal growth factor receptor, antibody-dependent cell cytotoxicity, complement-dependent cytotoxicity

## Abstract

The now clinically-used anti-epidermal growth factor receptor (EGFR) monoclonal antibodies have demonstrated significant efficacy only in patients with metastatic colorectal cancer (mCRC), with wild-type Kirsten rat sarcoma viral oncogene homolog (*KRAS*). However, no effective treatments for patients with mCRC with *KRAS* mutated tumors have been approved yet. Therefore, a new strategy for targeting mCRC with *KRAS* mutated tumors is desired. In the present study, we examined the anti-tumor activities of a novel anti-EGFR monoclonal antibody, EMab-17 (mouse IgG_2a_, kappa), in colorectal cancer (CRC) cells with the *KRAS* p.G13D mutation. This antibody recognized endogenous EGRF in CRC cells with or without *KRAS* mutations, and showed a high sensitivity for CRC cells in flow cytometry, indicating that EMab-17 possesses a high binding affinity to the endogenous EGFR. In vitro experiments showed that EMab-17 exhibited antibody-dependent cellular cytotoxicity and complement-dependent cytotoxicity activities against CRC cells. In vivo analysis revealed that EMab-17 inhibited the metastases of HCT-15 and HCT-116 cells in the livers of nude mouse metastatic models, unlike the anti-EGFR monoclonal antibody EMab-51 of subtype mouse IgG_1_. In conclusion, EMab-17 may be useful in an antibody-based therapy against mCRC with the *KRAS* p.G13D mutation.

## 1. Introduction

The epidermal growth factor receptor (EGFR) is a member of the ErbB family of receptors, a subfamily of four receptor tyrosine kinases, namely EGFR (Erb-1), human EGFR (HER)2 (Erb-2), HER3 (Erb-3) and HER4 (Erb-4). It regulates cell proliferation, survival, differentiation and migration [1]. Ligand binding to the extracellular part of EGFR causes receptor dimerization, leading to the activation of the downstream signaling of the Mitogen-activated protein kinases (MAPK)/Extracellular signal-regulated kinase (ERK) and phosphoinositide 3-kinase (PI3K)/Akt pathways, and influences gene transcription [2,3,4]. Numerous tumors, including head and neck [5], lung [6], pancreas [7], colon [8], breast [9], kidney [10], prostate and bladder cancers [11], overexpress EGFR. In addition, the dysregulation of EGFR signaling is associated with poor prognosis [12], rendering this receptor a potential target for anti-tumor treatment.

Metastatic colorectal cancer (mCRC) is one of the most aggressive malignancies with high mortality rates worldwide [13]. The liver is well recognized as the most frequent metastatic site of colorectal cancer (CRC), and CRC liver metastasis is highly associated with poor prognosis and low patient survival. Immunohistochemical analysis revealed that EGFR is overexpressed in many patients with CRC, making EGFR an attractive therapeutic option [14]. In contrast, approximately 30–50% of patients with CRC harbor Kirsten rat sarcoma viral oncogene homolog (*KRAS*) mutations. Although the anti-EGFR antibodies cetuximab and panitumumab are used to treat mCRC, their use is limited to patients with wild-type *KRAS*, because patients with mutations in *KRAS* exon 2 (codon 12 or 13) do not benefit from anti-EGFR treatment [15,16]. The most frequent mutations occur in exon 2 (codon 12—p.G12D, 13%, and p.G12V, 9%; and codon 13—p.G13D, 8%) of *KRAS* [17]. However, there are conflicting reports with respect to mutations in codon 13 (p. G13D) of *KRAS*. Several retrospective analyses found that cetuximab provides increased clinical benefits to patients with mCRC with *KRAS* p.G13D mutations over those with other *KRAS* mutations [17,18]. Cells from *KRAS* p.G12V-mutated tumors were not responsive to cetuximab, whereas cells from *KRAS* p.G13D-mutated tumors were as responsive to cetuximab as *KRAS* wild-type cells [19]. However, patients with mCRC with *KRAS* p.G13D mutations were not shown to benefit from panitumumab therapy in three randomized phase III trials [20]. Owing to the limitations of retrospective studies and the low number of patients with *KRAS* mutations in the datasets, further clinical studies with larger sample sizes are required in order to evaluate the differences in the efficacy of the EGFR-targeting strategy for mCRC with *KRAS* p.G13D mutation and for that with *KRAS* mutations other than p.G13D.

Recently, we developed a novel anti-EGFR mAb (EMab-17, IgG_2a_, kappa) by immunizing mice with an EGFR-overexpressing glioblastoma cell line, LN229 (LN229/EGFR) [21]. EMab-17 showed antibody-dependent cellular cytotoxicity (ADCC) and complement-dependent cytotoxicity (CDC) activity against two human oral squamous cell carcinoma (OSCC) cell lines, HSC-2 and SAS. EMab-17 also exhibited anti-tumor activity in mouse xenograft models of OSCC [21]. These results suggest that EMab-17 can be used as an effective treatment for OSCC. On the basis of the effects of EMab-17 against OSCC cell lines, we explored whether EMab-17 shows similar anti-tumor activity in CRC cell lines with *KRAS* p.G13D mutations, as there are few effective treatments for patients with mCRC with *KRAS* mutated tumors. EMab-17 exhibited both anti-tumor and anti-metastatic activities in xenograft models of CRC.

## 2. Results

### 2.1. Growth Inhibitory Activity of EMab-17 in Xenograft Models

**Figure 1:**
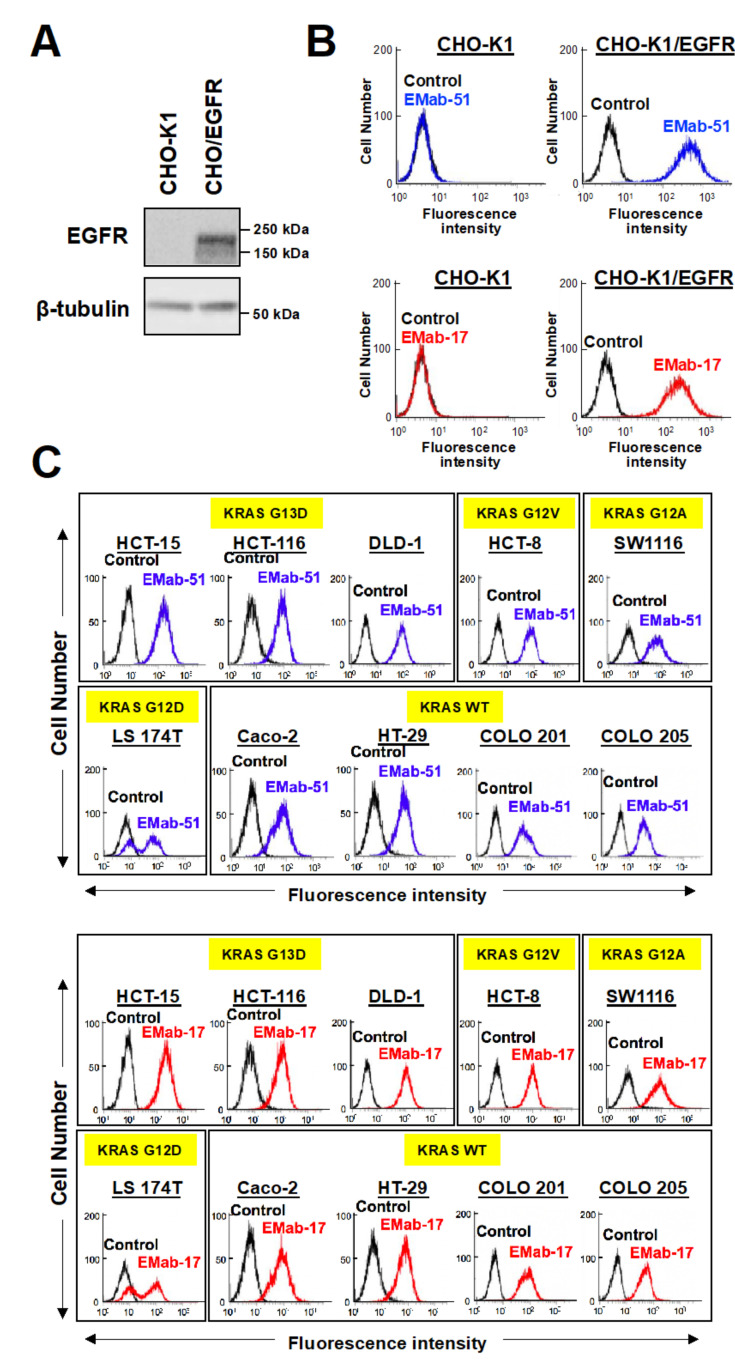
Flow cytometry of epidermal growth factor receptor (EGFR)-transfected and EGFR-expressing cell lines using anti-EGFR antibodies EMab-17 and EMab-51. (**A**) CHO-K1 and CHO/EFGR cells were subjected to Western blotting analysis with an anti-EGFR or anti-β-tubulin antibody. (**B**) CHO-K1 and EGFR-transfected CHO-K1, CHO/EGFR cells were treated with 1 μg/mL of anti-EGFR antibodies, EMab-51 (**Left**; blue) and EMab-17 (**Right**; red). After treatment with the anti-EGFR antibodies, the cells were treated with Alexa Fluor 488-conjugated anti-mouse IgG; black line, negative control. Fluorescence data were collected using a Cell Analyzer EC800. (**C**) CRC cell lines with or without *KRAS* mutations were treated with EMab-51 (**Top**; blue) and EMab-17 (**Bottom**; red). Yellow highlights indicate the KRAS status of CRC cell lines. Data were analyzed as in (**B**).

Anti-EGFR mAbs have been used as an effective first-line treatment for patients with mCRC. Therefore, we characterized EMab-17 using CHO-K1 and CHO/EGFR cells (Figure 1A). EMab-17 reacted with CHO/EGFR but not with CHO-K1 by flow cytometry (Figure 1B), indicating that it is specific for EGFR. EMab-51 of subtype IgG_1_ (positive control) also reacted in the same pattern (Figure 1B) [22].

**Figure 2:**
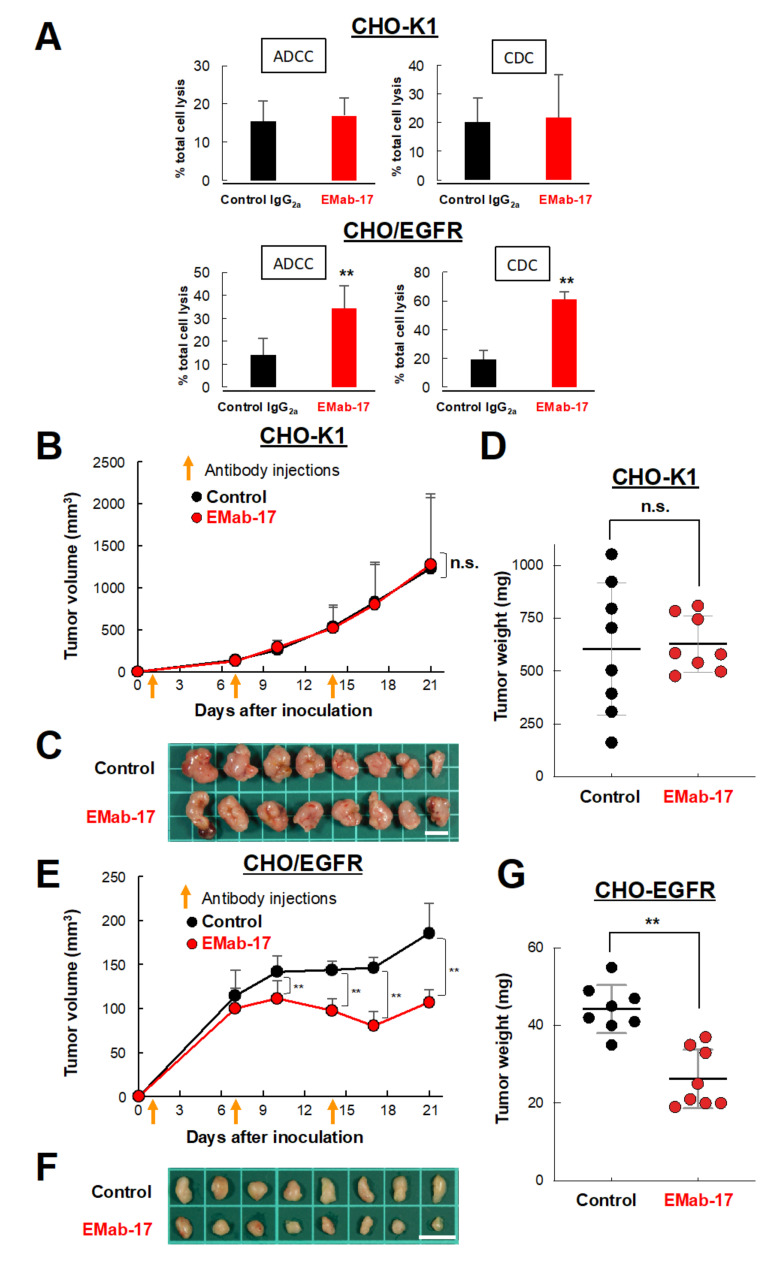
Antibody-dependent cellular cytotoxicity (ADCC), complement-dependent cytotoxicity (CDC) and anti-tumor activities of EMab-17 against CHO/EGFR cells. (**A**) ADCC (**Left**) and CDC (**Right**) activities of EMab-17 against CHO-K1 (**Top**) or CHO/EGFR (**Bottom**) in vitro. ADCC activity was evaluated through the calcein-AM release assay in the presence of antibodies (100 μg/mL; effector/target ratio, 50). CDC activity was determined using the MTS assay in the presence of antibodies (100 μg/mL) or control mouse IgG_2a_ (100 μg/mL) with 10% rabbit complement. **: *p* < 0.01 vs. IgG_2a_-treated control. (**B**, **E**) Anti-tumor activity of EMab-17 against CHO-K1 or CHO/EGFR cells in vivo. Tumor volume of CHO-K1 (**B**) or CHO/EGFR (**E**) xenografts. Cells (5 × 10^6^ cells/100 μL) were subcutaneously inoculated into BALB/c nude mice. After 1 day, 100 μg of EMab-17 or control mouse IgG were injected into the peritoneal cavities of the mice. The orange arrows indicate the days of antibody injection and the antibodies were injected thrice (days 1, 7 and 14; control: *n* = 8; EMab-17: *n* = 8). The tumor diameter was measured at the indicated days and calculated using the formula: tumor volume = W^2^ × L/2, where W is the short diameter and L is the long diameter. Values are presented as means (SD). n.s.: not significant, **: *p* < 0.01 vs. control. (**C**, **F**) Resected tumors of CHO-K1 (**C**) or CHO/EGFR (**F**) xenografts on day 21. Scale bar: 10 mm. (**D**, **G**) Tumor weight of CHO-K1 (**D**) or CHO/EGFR (**G**) xenografts. Values are presented as means ± SEM. n.s.: not significant, **: *p* < 0.01 vs. control.

We subsequently evaluated the ADCC and CDC activities of EMab-17. Using calcein-AM, we labeled CHO-K1 and CHO/EGFR cells as target cells, and used splenocytes derived from BALB/c nude mice as a source of effector cells. As a result, we found that EMab-17 significantly augmented ADCC and CDC activities against CHO/EGFR cells (Figure 2A). In addition, a xenograft model was used to further examine the growth inhibitory activity of EMab-17 in vivo. CHO-K1 or CHO/EGFR cells were subcutaneously implanted into the flanks of nude mice at the age of 7 weeks. EMab-17 and control mouse IgG were injected thrice (on days 1, 7 and 14 after injection of cells) into the peritoneal cavities of mice. Cell growth was observed in mice from the control IgG-treated and EMab-17-treated groups in both CHO-K1 and CHO/EGFR xenograft models. As expected, EMab-17 significantly inhibited cell growth in the CHO/EGFR xenografts compared to the control mouse IgG on days 10, 14, 17 and 21 (Figure 2E,F). Additionally, the cell weight in the EMab-17-treated group was significantly lower than that in the control IgG-treated group (Figure 2G). In the CHO-K1 xenograft model, there was no difference in cell growth and weight between control IgG-treated and EMab-17-treated groups (Figure 2B–D). Mouse body weight was not significantly different among the two groups in the CHO-K1 or CHO/EGFR xenograft models (Figure S1). These results suggest that EMab-17 is sensitive and specific against EGFR.

### 2.2. Anti-Tumor Activity of EMab-17 against CRC Cells with the KRAS p.G13D Mutation

To characterize the reactivity of EMab-17 to CRC cell lines, we used CRC cells with *KRAS* p.G13D (HCT-15, HCT-116, DLD-1), *KRAS* p.G12V (HCT-8), *KRAS* p.G12A (SW1116), *KRAS* p.G12D (LS174T) and *KRAS* wild-type (Caco-2, HT-29, COLO201, COLO205). As shown in Figure 1C, EMab-17 recognized endogenous EGRF in CRC cells with or without *KRAS* mutations using flow cytometry, and EMab-51 showed the same reaction as that of EMab-17.

**Figure 3:**
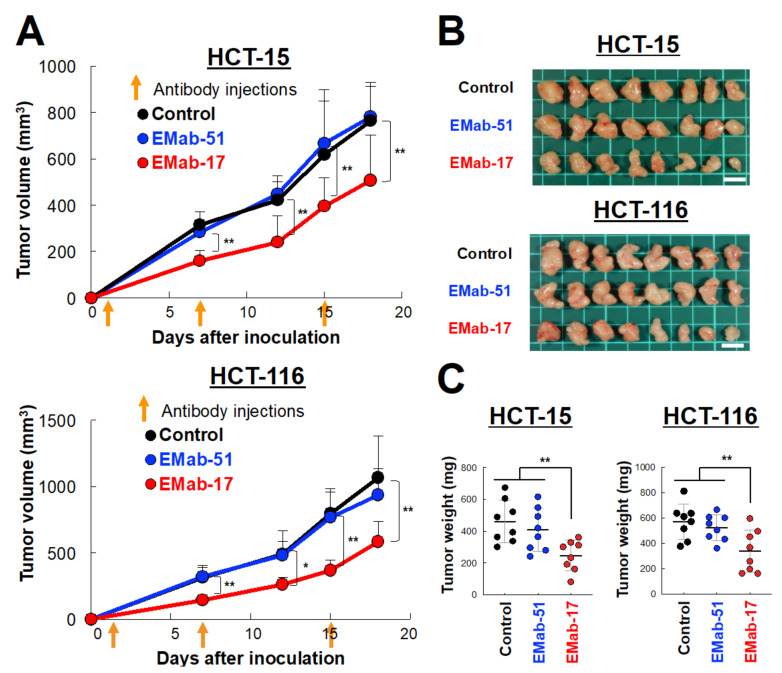
EMab-17 inhibits the growth of colorectal cancer (CRC) cell lines with *KRAS* p.G13D mutation. (**A**) Anti-tumor activity of EMab-17 against HCT-15 (**Top**) and HCT-116 (**Bottom**) cells in vivo. Tumor volume of xenografts treated with mouse control IgG, EMab-17 or EMab-51. Cells (5 × 10^6^ cells/100 μL) were subcutaneously inoculated into BALB/c nude mice. After 1 day, 100 μg of EMab-51, EMab-17 or control mouse IgG were injected into the peritoneal cavities of the mice. The orange arrows indicate the days of antibody injection and the antibodies were injected thrice (days 1, 7, and 15; control: *n* = 8; EMab-51: *n* = 8, EMab-17: *n* = 8). Values are presented as means (SD). *: *p* < 0.05, **: *p* < 0.01 vs. control. (**B**) Resected tumors of HCT-15 or HCT-116 xenografts on day 18. (**C**) Tumor weight of HCT-15 (**Left**) or HCT-116 (**Right**) xenografts. Scale bar: 1 cm. Values are presented as means ± SEM. **: *p* < 0.01 vs. control and EMab-51-treated groups.

Next, we investigated whether EMab-17 inhibited the growth of *KRAS* p.G13D-mutated CRC cells in vivo. CRC cell lines with *KRAS* p.G13D (HCT-15 and HCT-116) were subcutaneously inoculated into the right flank of each nude mouse to establish a system for examining the anti-tumor activity of EMab-17 in xenograft models. For this, 100 μg doses of either EMab-17, EMab-51 or control antibodies were injected intraperitoneally (i.p.) once a week for 3 weeks on days 1, 7 and 15 after the injection of cancer cells. Compared with the control IgG treatment, EMab-17, unlike EMab-51, significantly reduced tumor development in both HCT-15 and HCT-116 cells on days 7, 12, 15 and 18 (Figure 3A). The mice were sacrificed 18 days after the injection of cancer cells, and tumors were removed and weighed (Figure 3B). The weights of the tumors of the EMab-17-treated mice were significantly lower than those of the control IgG- and EMab-51-treated mice (Figure 3C). Body weight was not significantly different among the three groups (Figure S2). Furthermore, we performed immunohistochemical analysis of the isolated tumor tissues (Day 18) using the proliferation marker, and investigated the relationship between the proportion of Ki-67 positive cells and the anti-tumor activity of EMab-17. As shown in Figure S3A,B, EMab-17-treated tumors exhibited a significantly reduced expression of Ki-67, unlike EMab-51-treated tumors. These results suggested that EMab-17 exhibited anti-tumor activity against *KRAS* p.G13D-mutated CRC cells in vivo. To examine the possibility that EMab-17 inhibits tumor growth in xenograft models, we performed in vitro experiments. EGFR signaling is initiated by the ligand binding of epidermal growth factor (EGF) family members to EGFR, leading to receptor dimerization and the subsequent activation of growth signals [2,4]. When EGF was added, HCT-15, HCT-116 and DLD-1 cells did not proliferate well compared with the control cells, and did not respond to EGF with or without EMab-17 or EMab-51 (Figure S4). These results suggest that EMab-17 does not neutralize the EGRF-driven cell proliferation, but exerts significant anti-tumor activity against CRC cells with the *KRAS* p.G13D mutation.

### 2.3. Determination of the Binding Affinity of EMab-17 against CRC Cell Lines with KRAS p.G13D Mutation

**Figure 4:**
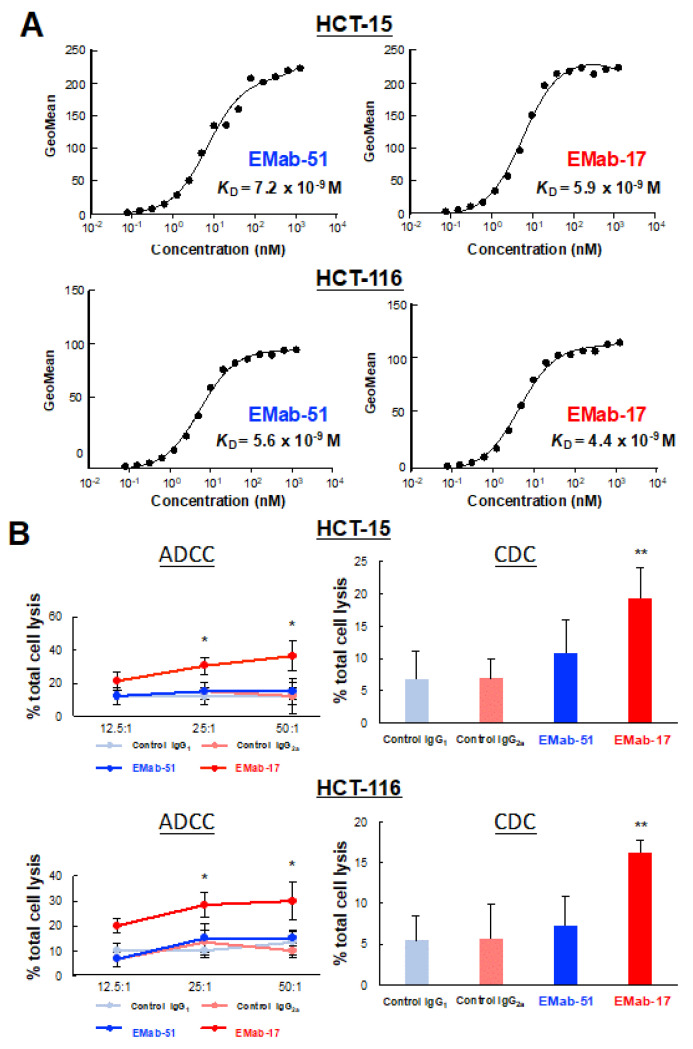
Determination of the binding affinity and ADCC and CDC activities of EMab-17 or EMab-51 using flow cytometry. (**A**) HCT-15 (**Top**) and HCT-116 (**Bottom**) cells were suspended in 100 μL of serially diluted EMab-17 or EMab-51 (6 to 100 ng/mL), followed by the addition of secondary anti-mouse IgG. Fluorescence data were collected using a cell analyzer. (**B**) The ADCC and CDC activities of EMab-17 or EMab-51 against HCT-15 (**Top**) or HCT-116 (**Bottom**) cells were evaluated. ADCC activity was evaluated through the calcein-AM release assay in the presence of antibodies (100 μg/mL; effector/target ratio, 12.5, 25 and 50). CDC activity was determined using the MTS assay in the presence of antibodies (100 μg/mL) or control mouse IgGs (IgG_1_ or IgG_2a_, 100 μg/mL) with 10% rabbit complement. The values are means (SD). *: *p* < 0.05, **: *p* < 0.01 vs. IgG_2a_-treated control.

The binding affinity of antibodies is essential for antibody-based anti-tumor activity. Therefore, we performed a kinetic analysis of the interaction of EMab-17 and EMab-51 with CRC cells with *KRAS* p.G13D mutation. As shown in Figure 4A, the apparent dissociate constant (*K*_D_) of EMab-51 was 7.2 × 10^−9^ M for HCT-15 and 5.6 × 10^−9^ M for HCT-116, respectively. Similarly, EMab-17 showed high binding affinity (5.9 × 10^−9^ M for HCT-15 and 4.4 × 10^−9^ M for HCT-116).

### 2.4. EMab-17 Exerts ADCC and CDC Activities against CRC Cell Lines with KRAS p.G13D Mutation

Because EMab-17 showed high binding affinity and anti-tumor activity against HCT-15 and HCT-116 cells, we evaluated the ADCC and CDC activities of EMab-17 for CRC cell lines with *KRAS* p.G13D mutation. As expected, EMab-17, unlike EMab-51, significantly augmented ADCC activity against both HCT-15 and HCT-116 cells. In addition, EMab-17, unlike EMab-51, significantly enhanced CDC activity against HCT-15 and HCT-116 cells following the addition of the rabbit complement (Figure 4B). Additionally, EMab-17, unlike EMab-51, also significantly augmented ADCC and CDC activities against DLD-1 (Figure S5). These results indicate that EMab-17 exerts anti-tumor activity through both ADCC and CDC activities.

### 2.5. Characterization of a Metastatic Model of CRC Cells with KRAS p.G13D Mutation

The liver is known to be the most frequent site of metastasis of CRC [23]. To examine the effect of antibodies on the capacity of CRC to metastasize to the liver, we investigated EGFP-expressing CRC cell lines with the *KRAS* p.G13D mutation (DLD-1, HCT-15, HCT-116) using a CRC liver metastasis mouse model created by intrasplenic injection [24,25]. CRC cells (2.5 × 10^5^ cells suspended in Matrigel/10 μL) were injected into the spleen of six-week-old female nude mice. The mice were euthanized 26 or 27 days after cell implantation. In this model, DLD-1 (0/3) failed to metastasize to the liver, whereas HCT-15 (3/3) and HCT-116 (3/3) resulted in liver metastases (Figure S6A–C).

**Figure 5:**
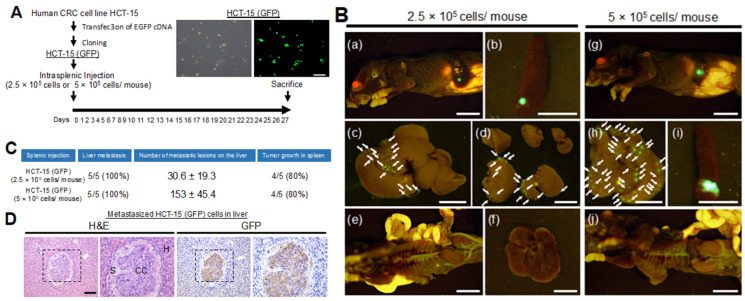
Characterization of a model of CRC metastasis to the liver in nude mice. (**A**) The experimental schedule. Cloned GFP-labeled HCT-15 (HCT-15-GFP) cells (2.5 × 10^5^ cells or 5 × 10^5^ cells suspended with Matrigel/10 μL) were injected into the spleens. Mice were sacrificed 27 days after the injection of cancer cells, and the livers were removed to determine the frequency of liver metastasis. (**Upper right**) The pictures of cloned HCT-15-GFP cells. Scale bar: 50 μm. (**B**) Growth of HCT-15-GFP cells in the injected spleens and formation of metastasis in livers. A total of 2.5 × 10^5^ cells (**left**) or 5 × 10^5^ cells (**right**) were injected into the spleen, and images were captured after sacrifice. Representative images of body (**a**, **g**), spleen (**b**, **i**), liver (**c**, **d**, **h**), abdominal cavity (**e**, **j**) and lung (**f**). The arrows point to the site of metastasis. Scale bar: 10 mm. (**C**) The status of the model is shown in the table. The number of metastatic lesions in the liver is shown as means ± SEM. The frequency of liver metastasis and tumor growth in the spleen are shown in brackets. (**D**) Representative histological appearance of metastasized HCT-15-GFP cells in the liver (**left**) and immunohistochemical detection of HCT-15-GFP cells. Scale bar: 100 μm.

We optimized the CRC liver metastasis mouse model using different numbers of HCT-15-GFP cells. We injected HCT-15-GFP cells (2.5 × 10^5^ cells or 5 × 10^5^ cells suspended in Matrigel/10 μL) into the spleens of mice. We found that both cell concentrations could produce liver metastases in a cell number-dependent manner (2.5 × 10^5^ cells/mouse: 30.6 ± 19.3 per liver or 5 × 10^5^ cells: 153 ± 45.4 per liver) (Figure 5A–C). Using immunohistochemical analysis, we confirmed that the cells in the injected spleen and metastasized liver were GFP positive (Figure 5D). However, we did not detect metastasis in other organs (i.e., lung, kidney and intestine) (Figure 5B). Intriguingly, we found that some mice form liver metastasis without growth of cells in the injected spleen (Figure 5C). These results suggest that HCT-15 and HCT-116 cell lines may be used to examine the effect of EMab-17 on the liver metastasis of CRC cells with *KRAS* p.G13D mutation.

### 2.6. Anti-Metastatic Activity of EMab-17 in Mouse Liver Suppression of CRC Cells with KRAS p.G13D Mutation Metastasis to the Liver by EMab-17

**Figure 6:**
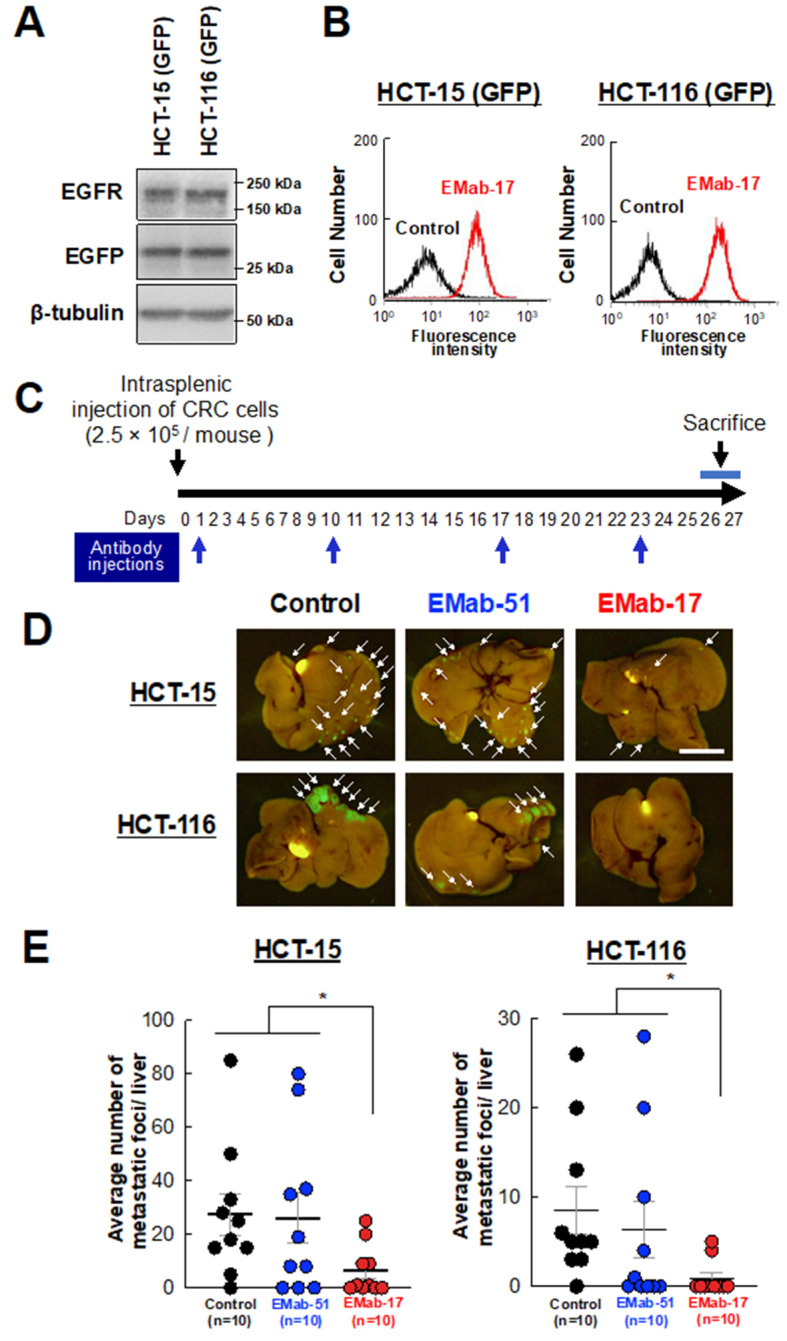
Anti-metastatic effects of an anti-EGFR antibody EMab-17 on CRC metastasis to the liver. (**A**) HCT-15-GFP and cloned GFP-labeled HCT-116 (HCT-116-GFP) cells were subjected to Western blotting analysis with an anti-EGFR, anti-GFP or anti-β-tubulin antibody. (**B**) Flow cytometry using the anti-EGFR antibody EMab-17 for HCT-15-GFP or HCT-116-GFP cells. (**C**) Experimental schedule. HCT-15-GFP and HCT-116-GFP cells (2.5 × 10^5^ cells suspended with Matrigel/10 μL) were injected into the spleens. The antibodies (100 μg/mouse) were intraperitoneally administered 1, 10, 17 and 23 days after the inoculation of cancer cells. The mice were sacrificed 26 or 27 days after the injection of cancer cells, and the livers were removed to determine the number of metastatic CRC cells. (**D**) Representative images of the liver. The arrows point to the site of metastasis. Scale bar: 1 cm. (**E**) Number of metastatic tumors in the liver. The mean number for each group is expressed with a horizontal bar; 10 mice were used per group. The values are presented as means ± SEM. *: *p* < 0.05 vs. control and EMab-51-treated groups.

Next, we investigated whether EMab-17 reacts with EGFP-expressing HCT-15 and HCT-116 cells (Figure 6A and Figure S7). As shown in Figure 6B, EMab-17 reacted with both cell lines at a low concentration of 1 μg/mL. We then investigated the anti-metastatic activity of EMab-17 using the mouse model of CRC metastasis to the liver with GFP-labeled HCT-15 and HCT-116 cells. EMab-17, EMab-51 and mouse control IgG were injected, once a week for four weeks (on days 1, 10, 17 and 23 after intrasplenic injection of cancer cells), into the peritoneal cavity of mice. Mice were sacrificed 26 or 27 days after the cancer cells injection, all liver lobes were resected, and the total numbers of liver metastases were determined (Figure 6C). Compared with the control IgG treatment, EMab-17, unlike EMab-51, significantly reduced the number of liver metastases of HCT-15-GFP and HCT-116-GFP cells (Figure 6D,E). Body weight was not significantly different among the three groups (Figure S8). In addition, we performed an in vitro invasion assay after treating the cells with control IgG or EMab-17 and complement. As shown in Figure S9, EMab-17 suppressed the invasion activity of HCT-15 and HCT-116 cells compared with the control cells. These results suggest that EMab-17 can inhibit the metastasis of CRC to the liver through ADCC and CDC activities (Figure 6E).

## 3. Discussion

In this study, we examined whether a novel anti-EGFR mAb EMab-17 is useful for the treatment of mCRC with *KRAS* p.G13D mutation via ADCC and CDC activities, rather than via neutralization of the EGFR signaling to promote EGF-dependent cell proliferation, as CRC cells with *KRAS* mutations bypass the EGFR pathway. ADCC and CDC activities are considered important mechanisms of action in cancer immunotherapy. Human IgG contains four subclasses (IgG_1_, IgG_2_, IgG_3_ and IgG_4_), also termed isotypes, and has evolved different fragment crystallizable (Fc) sequences with differential activities to elicit effector functions. The human IgG_1_ elicits strong effector functions like ADCC and CDC [26]. In fact, the chimeric human/mouse IgG_1_ subclass mAb cetuximab can effectively elicit ADCC and CDC. On the other hand, the mouse IgG also contains four subclasses (IgG_1_, IgG_2a_, IgG_2b_ and IgG_3_). The mouse IgG_1_ subclass exhibits no or low CDC and no ADCC. In contrast, the mouse IgG_2a_ subclass is equivalent to human IgG_1_ and can elicit ADCC and CDC activities [27]. We have previously reported that EMab-51 belongs to the mouse IgG_1_ subclass [22]. EMab-17 belongs to the mouse IgG_2a_ subclass, and showed ADCC and CDC activities against oral squamous cell carcinomas [21]. On the basis of these results, we examined whether EMab-17 exerts similar anti-tumor activities against EGFR-expressing cells, especially CRC cells.

Two anti-hEGFR mAbs, cetuximab (chimeric human/mouse IgG_1_) and panitumumab (human IgG_2_), have been approved for the treatment of CRC with *KRAS* wild-type tumors [16,28]. Cetuximab and panitumumab recognize and bind to domainIII of EGFR, and show similar benefits and safety profiles [29]. Cetuximab shows activity after panitumumab failure, and panitumumab is effective after cetuximab failure, indicating that there are differences in the mechanisms of action of the two drugs, and more anti-EGFR agents are needed [30,31]. However, effective anti-EGFR agents for mCRC patients with *KRAS*-mutated tumors have not yet been approved. Here, we describe EMab-17 as a new candidate therapeutic mAb for mCRC patients with *KRAS* mutated tumors.

Targeted therapy using mAbs has been established as a powerful immunotherapy against many tumors. Because *KRAS* is a downstream effector of EGFR signaling, *KRAS* mutated CRC cells are thought to be resistant to anti-EGFR mAbs, due to the constitutive activation of *KRAS* signaling [32,33]. In this study, we found that the *KRAS* p.G13D mutant cell lines HCT-15, HCT-116 and DLD-1 were resistant to EMab-17 in vitro (Figure S4). In addition to the direct inhibition of EGFR signaling, ADCC and CDC activities are known to be an important mode of action of cetuximab [34]. ADCC and CDC are activated by the interaction of the Fc portion of the mAb with cytotoxic effector cells, such as natural killer cells, and complement the antibody. Since EMab-17 showed growth inhibitory activity in vivo, the induction of ADCC and CDC activities could be one of their modes of action.

Metastatic CRC with *KRAS* mutations is rarely curable, and the development of new effective therapeutic options is urgently warranted. Therefore, in the present study, we focused on the potential therapeutic use of EMab-17 in this setting. We characterized a metastatic model of CRC to the liver using nude mice through intrasplenic injection of tumor cells. Such mouse models have been previously reported [24,25,35]. We injected EGFP-expressing CRC cells with Matrigel to visualize metastatic CRC cells and precisely determine the number of metastatic CRC cells in the liver. Using this model, we examined which *KRAS* p.G13D-mutated CRC cells possess metastatic activity towards the liver. The results showed that EGFR-expressing HCT-15 and HCT-116 cells could successfully metastasize to the liver (Figure S6). Therefore, we used HCT-15 and HCT-116 cells to examine whether EMab-17 could suppress the metastasis of CRC to the liver. As shown in Figure 6D,E, EMab-17, unlike EMab-51, significantly reduced the number of *KRAS* p.G13D-mutated CRC cells metastasized to the liver. However, EMab-17 could not inhibit or diminish the presence of all CRC cells in the liver. As previously reported by this and other research groups, afucosylation of the Fc domain to increase the ADCC, and/or the combination of EMab-17 with anti-cancer drugs, are needed to enhance the anti-metastatic activity of EMab-17 [36,37,38,39]. In future studies, we need to generate a human/mouse chimeric anti-EGFR antibody from EMab-17 in order to develop the therapeutic antibody.

In this study, we evaluated the anti-EGFR mAb EMab-17. We found that it had ADCC- and CDC-inducing activities, leading to the suppression of the growth and metastasis of CRC cell lines with *KRAS* p.G13D. Recently, McFall et al. proposed a mechanism to explain why CRC cells with *KRAS* p.G13D retain sensitivity to anti-EGFR Abs [40]. Further studies into anti-tumor activities against EGFR-expressing CRC cells are necessary in order to obtain a more detailed understanding of antibody therapy against CRC cells with *KRAS* p.G13D, which will lead to the development of more effective clinical treatments.

## 4. Materials and Methods

### 4.1. Cell Lines

Chinese hamster ovary-K1 (CHO-K1), Caco-2, HCT-116, HCT-15, HT-29, LS 174T, COLO 201, COLO 205, HCT-8, SW1116 and DLD-1 were obtained from American Type Culture Collection (ATCC; Manassas, VA, USA). CHO/EGFR and LN229/EGFR cells were established in our previous study [22]. P3U1, CHO-K1 and CHO/EGFR were cultured in RPMI 1640 medium (Nacalai Tesque, Inc., Kyoto, Japan). LN229, LN229/HER2, Caco-2, HCT-116, HCT-15, HT-29, LS 174T, COLO 201, COLO 205, HCT-8, SW1116 and DLD-1 were cultured in DMEM (Nacalai Tesque, Inc.) supplemented with 10% heat-inactivated FBS (Thermo Fisher Scientific Inc., Waltham, MA, USA), 100 units/mL of penicillin, 100 μg/mL of streptomycin and 0.25 μg/mL of amphotericin B (Nacalai Tesque, Inc.) at 37 °C in a humidified atmosphere containing 5% CO_2_.

### 4.2. Western Blot Analysis

Western blot analysis was performed as described previously [41]. Briefly, the cells were lysed for 30 min on ice in lysis buffer (20 mmol/L HEPES pH 7.5; 150 mmol/L NaCl; 1% (v/v) Triton X-100; 10% (v/v) glycerol; 1 mmol/L EDTA; 50 mmol/L NaF; 50 mmol/L β-glycerophosphate; 1 mmol/L Na_3_VO_4_; and 25 μg/mL antipain, leupeptin and pepstatin). After centrifugation at 20,400× *g* for 10 min at 4 °C, the lysates were collected and separated by SDS-PAGE, transferred onto polyvinylidene difluoride membranes (Merck KGaA, Darmstadt, Germany), and subjected to Western blot. The protein levels were measured using the following primary antibodies: anti-EGFR antibody (#4267, 1:1000; Cell Signaling Technology, Inc., Danvers, MA, USA), anti-tubulin (#5346, 1:1000; Cell Signaling Technology) and anti-GFP antibody (ab6673, 1:500; Abcam, Cambridge, UK).

### 4.3. Animals

Mice were maintained in a pathogen-free environment on a 11 h light/13 h dark cycle at a temperature of 23 °C ± 2 °C and 55% ± 5% humidity with food and water supplied ad libitum throughout the experimental period. All animal experiments were approved by the Institutional Committee for Animal Experiments at the Institute of Microbial Chemistry (Permit no. 2019-014, 2019-069 for anti-tumor experiments, 2019-049, 2020-020 for ADCC assays, and 2019-049 for anti-metastatic experiments).

### 4.4. Growth Inhibitory Activity of EMab-17 In Vivo

Seven-week-old mice were used to study the growth inhibitory activity of EMab-17 in vivo. CHO/EGFR or CHO cells (0.3 mL of 1.33 × 10^8^/mL in RPMI) were mixed with 0.5 mL of Matrigel (BD Biosciences). A 100 μL suspension (containing 5 × 10^6^ cells) was subcutaneously injected into the left flanks of nude mice. After day 1, 100 μg of EMab-17 and control mouse IgG (I8765; Sigma-Aldrich Corp., St. Louis, MO, USA) in 100 μL PBS were injected into the peritoneal cavity of each mouse. Additional antibodies were injected on days 7 and 14. The tumor diameter and volume were determined as previously described [38]. The mice were euthanized 21 days after cell implantation. All data were expressed as mean ± SEM. Statistical analysis was performed using the two-tailed Student’s *t*-test. *p* < 0.05 denoted statistical significance.

### 4.5. Antibody-Dependent Cellular Cytotoxicity (ADCC) and Complement-Dependent Cytotoxicity (CDC) Activities

ADCC activity was assessed as previously described [42]. The spleens of BALB/c nude mice were aseptically removed, and single cell suspensions were obtained by dispersing the spleens using a syringe and pressing through stainless a steel mesh. Erythrocytes were effectively lysed via 10 s exposure to ice-cold distilled water. Splenocytes were washed with RPMI1640 or DMEM and resuspended in RPMI1640 or DMEM with 10% FBS as effector cells. CHO-K1, CHO-EGFR, HCT-15 or HCT-116 target cells were labeled with 10 μg/mL calcein-acetoxymethyl (calcein-AM) (Thermo Fisher Scientific Inc.) and resuspended in RPMI 1640 (for CHO-K1 or CHO-EGFR cells) and DMEM (for HCT-15 or HCT-116 cells). Target cells (2 × 10^4^ cells/well) were placed in 96-well plates and mixed with effector cells, anti-EGFR antibodies, or control IgGs (IgG_1_, M7894 and IgG_2a_, M7769; Sigma-Aldrich Corp.). After 4 h of incubation, the release of calcein-AM in the supernatant from each well was measured. The fluorescence intensity was determined at an excitation wavelength of 485 nm and an emission wavelength of 538 nm using a microplate reader (Power Scan HT; Bio Tek Instruments, Winooski, VT, USA). Cytolytic activity (as % lysis) was calculated using the following formula: % lysis = (E−S)/(M−S) × 100 (where E is the fluorescence released in experimental cultures of target cells and effector cells; S is the spontaneous fluorescence released in cultures containing only target cells; *M* is the maximum fluorescence obtained by adding lysis buffer containing 0.5% Triton X-100, 10 mM Tris–HCl [pH 7.4], and 10 mM EDTA to lyse all cells).

For CDC activity, CHO-K1, CHO-EGFR, HCT-15 or HCT-116 cells were placed in 96-well plates 2 × 10^4^ cells/well in RPMI 1640 (for CHO-K1 or CHO-EGFR cells) and DMEM (for HCT-15 or HCT-116 cells) supplemented with 10% FBS. Subsequently, the cells were incubated with the anti-EGFR antibodies or control IgGs (IgG_1_, M7894 and IgG_2a_, M7769; Sigma-Aldrich Corp.) and 10% of rabbit complement (Low-Tox-M rabbit complement; Cedarlane Laboratories, Hornby, Ontario, Canada) for 4 h at 37 °C. The MTS [3-(4,5-dimethylthiazol-2-yl)-5-(3-carboxymethoxyphenyl)-2-(4-sulfophenyl)-2H-tetrazolium; inner salt] assay was performed using a CellTiter 96 AQueous assay kit (Promega, Madison, WI, USA) as previously described [43] to evaluate cell viability.

### 4.6. Anti-Tumor Activity of EMab-17 In Vivo

Nude mice were used to study the anti-tumor activity of EMab-17 in vivo. Cells (0.3 mL of 1.33 × 10^8^/mL in RPMI) were mixed with 0.5 mL of Matrigel (BD Biosciences). A 100-μL suspension (containing 5 × 10^6^ cells) was subcutaneously injected into the left flanks of nude mice. After day 1, 100 μg of EMab-51, EMab-17 and control mouse IgG (I8765; Sigma-Aldrich Corp., St. Louis, MO, USA) in 100 μL of PBS were injected into the peritoneal cavity of each mouse. Additional antibodies were injected once a week. The tumor diameter and volume were determined as previously described [38]. The tumor weight was measured at the end of the experiment. All data were expressed as mean ± SEM. Statistical analysis was performed using the two-tailed Student’s *t*-test. *p* < 0.05 denoted statistical significance.

### 4.7. Immunohistochemical Analysis

Immunohistochemical analysis was performed as previously described [41,44]. Briefly, paraffin-embedded mouse liver tissues were sectioned, and the sections were placed on microscope slides. The deparaffinized sections were boiled in buffered sodium citrate solution (0.01 mol/L, pH 6.0) for 10 min and subjected to immunohistochemical staining with anti-Ki67 antibody (ab16667, 1:200; Abcam) and anti-GFP antibody (ab6673, 1:400; Abcam) followed by horseradish peroxidase-linked secondary antibody for 30 min. The tissues were stained with 3,3′-diaminobenzidine using the ImmPACT DAB (Vector Laboratories, Burlingame, CA, USA) or the ChemMate EnVision Kit (Agilent Technologies, Inc., Santa Clara, CA, USA). Slides were immersed in hematoxylin for counterstaining and then observed under a Nikon Biophot microscope (Japan) and photographed using a digital camera (Nikon Digital Sight DS-Ri1, Tokyo, Japan).

### 4.8. Flow Cytometry

Cells were harvested by brief exposure to 0.25% trypsin/1 mM EDTA (Nacalai Tesque, Inc.). After washing with 0.1% bovine serum albumin (BSA)/PBS, the cells were treated with 1 μg/mL of anti-EGFR (EMab-17 and EMab-51) for 30 min at 4 °C and subsequently with Alexa Fluor 488-conjugated anti-mouse IgG (1:1000; Cell Signaling Technology, Inc.). Fluorescence data were collected using EC800 Cell Analyzers (Sony Corp., Tokyo, Japan).

### 4.9. Determination of the Binding Affinity Using Flow Cytometry

HCT-116 or HCT-15 cells (2 × 10^5^ cells) were suspended in 100 μL of serially diluted mAbs (0.6 ng/mL–10 μg/mL), followed by addition of Alexa Fluor 488-conjugated anti-mouse IgG (1:1000; Cell Signaling Technology, Inc.). Fluorescence data were collected using a cell analyzer (EC800; Sony Corp.). The dissociation constant (*K*_D_) was calculated by fitting the binding isotherms using the built-in one-site binding models in GraphPad PRISM 6 (GraphPad Software, Inc., La Jolla, CA, USA).

### 4.10. Characterization of the CRC Cells That Cause Liver Metastasis Using a Nude Mouse Model

All animal studies were approved by the Institutional Committee for Animal Experiments at the Institute of Microbial Chemistry and performed in accordance with relevant guidelines and regulations to minimize animal suffering. Five-week-old female BALB/C nu/nu mice were purchased from Charles River (Kanagawa, Japan). Six-week-old mice were used to characterize the CRC cells that cause CRC liver metastasis in vivo. Nude mice were anesthetized using ketamine-based anesthesia (100 mg/kg ketamine, 2.5 mg/kg xylazine and 2.5 mg/kg acepromazine), and green fluorescent protein (GFP)-labeled HCT-15 cells (0.3 mL of 1.33 × 10^8^/mL or 0.3 mL of 6.65 × 10^7^/mL in DMEM) were mixed with 0.5 mL of BD Matrigel Matrix Growth Factor Reduced (BD Biosciences, San Jose, CA, USA). A 10-μL suspension (containing 5 × 10^5^ cells or 2.5 × 10^5^ cells) was injected into the spleens of nude mice to produce liver metastasis and washed with saline to prevent leakage. The spleen was replaced, and the abdominal wall and skin were closed using wound clips. The mice were euthanized 26 or 27 days after cell implantation, and the OV110 was used to obtain fluorescence images (Olympus Corp., Tokyo, Japan). We obtained GFP-labeled CRC cell lines (DLD-1-GFP and HCT-116-GFP) to characterize this model using other CRC cells. The GFP-labeled cells (0.3 mL of 6.6 × 10^7^/mL in DMEM) were mixed with 0.5 mL of Matrigel. A 10-μL suspension (containing 2.5 × 10^5^ cells) was injected into the spleens of nude mice to produce liver metastasis and washed with saline to prevent leakage. The method was repeated as described above.

### 4.11. Anti-Metastatic Activity of EMab-17 In Vivo

Six-week-old mice were used to study the anti-metastatic activity of EMab-17 in vivo. Nude mice were anesthetized using ketamine-based anesthesia, and HCT-15-GFP or HCT-116-GFP cells (0.3 mL of 6.65 × 10^7^/mL in DMEM) were mixed with 0.5 mL of Matrigel (BD Biosciences). A 10-μL suspension (containing 2.5 × 10^5^ cells) was injected into the spleens of nude mice to produce liver metastasis and washed with saline to prevent leakage. The spleen was replaced, and the abdominal wall and skin were closed using wound clips. After day 1, 100 μg of EMab-51, EMab-17 and control mouse IgG (I8765; Sigma-Aldrich Corp.) in 100 μL of PBS were injected into the peritoneal cavity of each mouse. Additional antibodies were then injected on days 10, 17 and 23. The mice were euthanized 26 or 27 days after cell implantation, and the OV110 was used to obtain fluorescence images (Olympus). All data were expressed as mean ± SEM. Statistical analysis was performed using the two-tailed Student’s *t*-test. *p* < 0.05 denoted statistical significance.

### 4.12. In Vitro Invasion Assay

The invasion assay was performed using the Cytoselect 96-well collagen cell invasion assay kit (Cell Biolabs, San Diego, CA, USA) according to the manufacturer’s protocol. Briefly, HCT-15 or HCT-116 cells were placed in 6-well plates at a density of 5 × 10^5^ cells/well in DMEM supplemented with 10% FBS. Next, the cells were incubated with the anti-EGFR antibody (EMab-17) or control IgG (IgG_2a_) and 10% rabbit complement for 2 h at 37 °C. The cells were washed three times with serum-free DMEM and were then added (in serum-free DMEM) to the top insert, which contained a polycarbonate membrane with 8-μm pores coated with a layer of bovine type I collagen matrix. DMEM with 10% FBS was added to the lower chamber. The chambers were then placed in the CO_2_ incubator for 24 h. The cells that passed through the membrane were lysed and quantitated using a fluorescence dye-containing solution and a microplate reader (Power Scan HT).

### 4.13. Statistical Analysis

All statistical comparisons were completed by the two-tailed Student’s *t*-test using the GraphPad Prism 8 (GraphPad Software, Inc., La Jolla, CA, USA). *p* < 0.05 was considered as statistically significant.

## Supplementary Materials

Supplementary materials can be found at https://www.mdpi.com/1422-0067/21/17/6037/s1.

## Abbreviations

EGFR	Epidermal growth factor receptor
KRAS	Kirsten rat sarcoma viral oncogene homolog
mCRC	Metastatic colorectal cancer
CRC	Colorectal cancer
ADCC	Antibody-dependent cellular cytotoxicity
CDC	Complement-dependent cytotoxicity
MAPK	Mitogen-activated protein kinases
ERK	Extracellular signal-regulated kinase
PI3K	Phosphoinositide 3-kinase
OSCC	Oral squamous cell carcinoma
