# Supplementary material for: Anti-Metastatic Activity of an Anti-EGFR Monoclonal Antibody against Metastatic Colorectal Cancer with KRAS p.G13D Mutation
Source: Int J Mol Sci. 2020 Aug 21;21(17):6037. doi: 10.3390/ijms21176037 (PMC7504481; doi:10.3390/ijms21176037)
Supplement: Supplementary file 1 [file ijms-21-06037-s001.pdf]

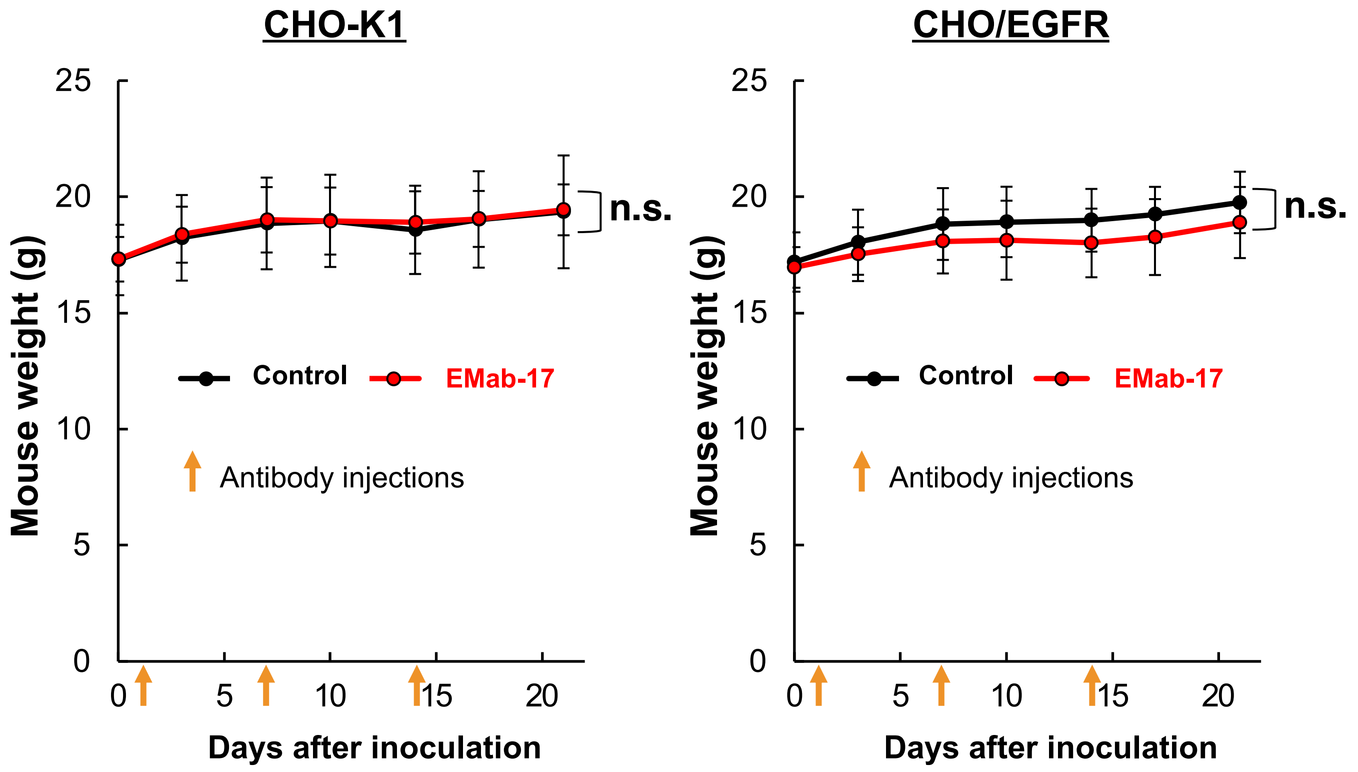

Figure S1 Body weight was not changed in mice treated with control IgG or EMab-17. Change in body weight was observed in the mice from Fig. 2B and E. n.s.: not significant vs. control.

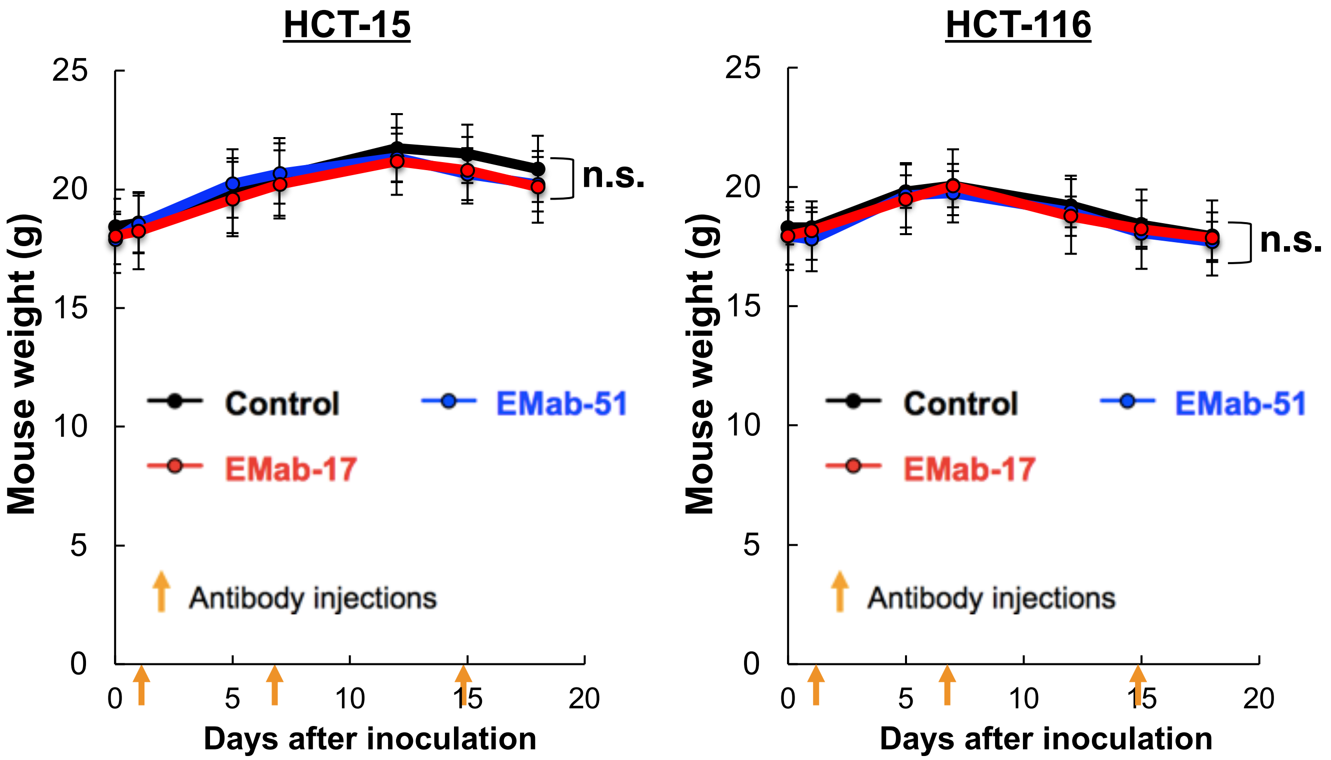

Figure S2 Body weight was not changed in mice treated with control IgG, EMab-51, or EMab-17. Change in body weight was observed in the mice from Fig. 3A. n.s.: not significant vs. control.

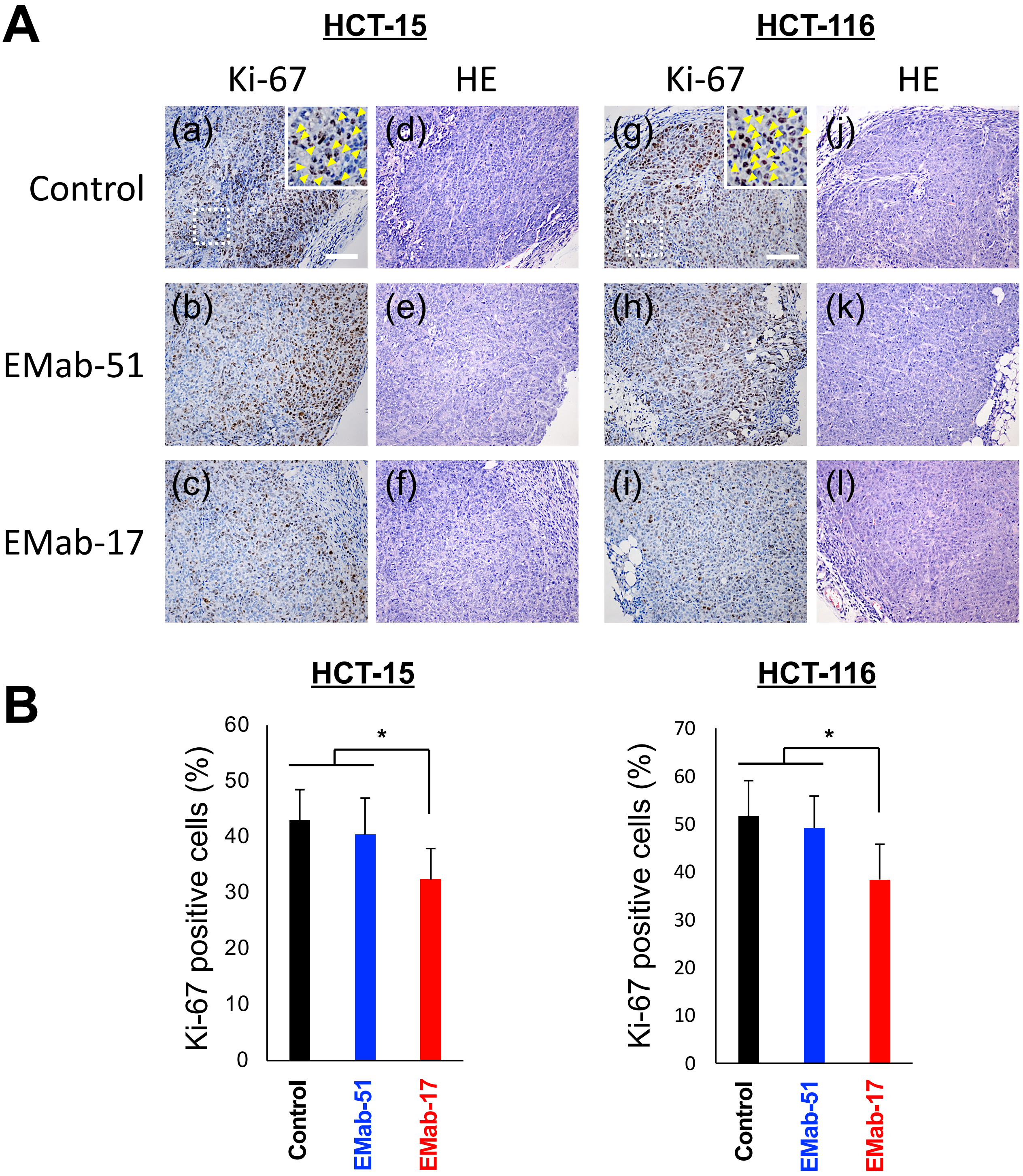

Figure S3 The Ki-67 positive cells determined by immunohistochemistry. (**A**) The expression of Ki-67 protein in tumor tissue was detected by immunohistochemistry (scale bar=100 μm). Tissue sectioned were incubated with Ki-67 antibody (a, b, c, g, h, i). Hematoxylin & eosin staining of Ki-67-positive cells (d, e, f, j, k, l). 6 random fields were selected from within each section. Values are presented as means (SD). *: $P < 0.05$ vs. control and EMab-51-treated groups.

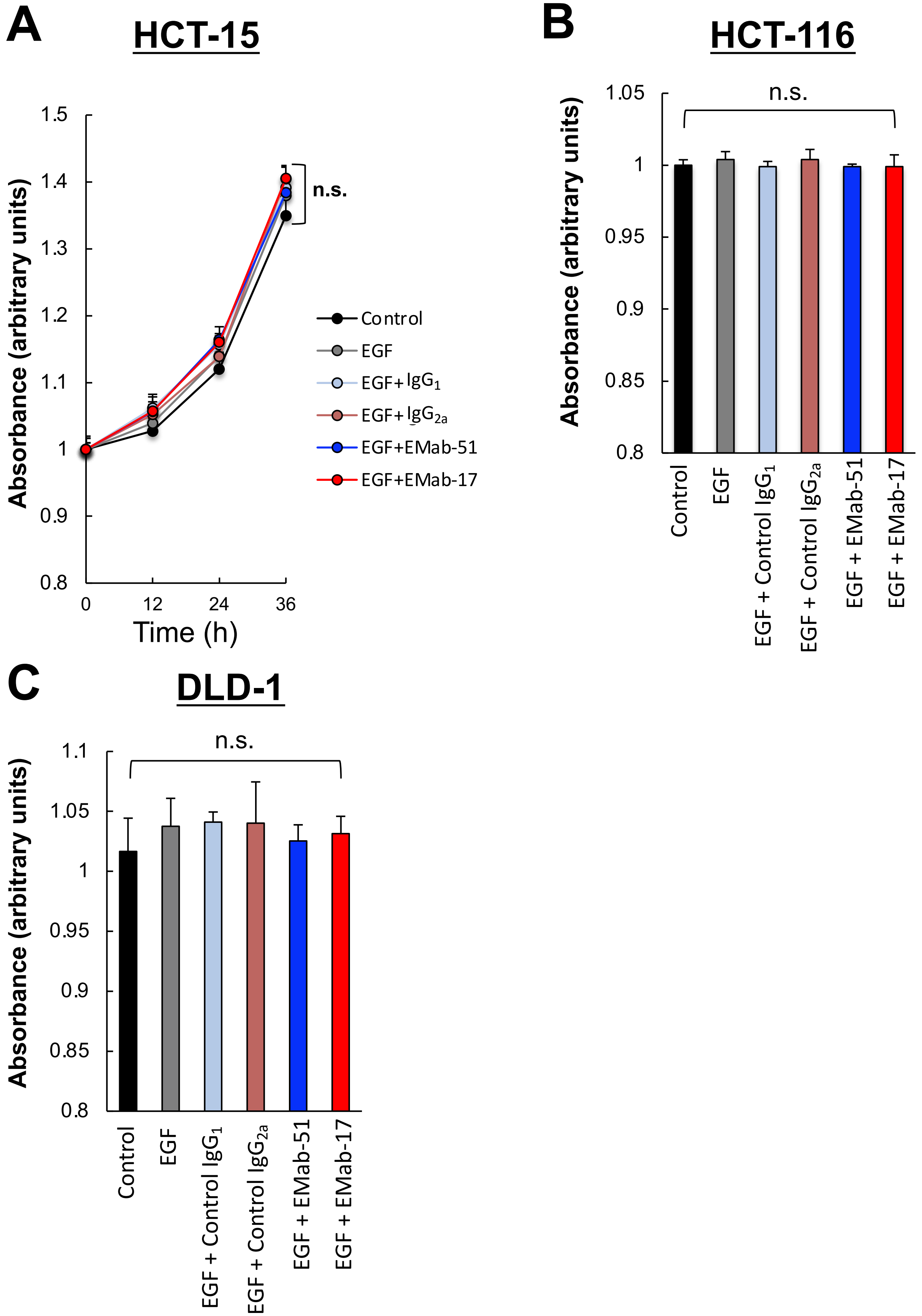

Figure S4 EGF did not stimulate the growth of HCT-15 and HCT-116 cells with or without EMab-17 and EMab-51. (A) HCT-15 cells were treated for 12–36 h with or without 0.5 ng/mL EGF or EGF + indicated IgGs (20 µg/mL). Cell growth was assayed using MTS assays. Values shown are mean (SD). n.s.: not significant vs. control. (B) HCT-116 cells were treated for 36 h with or without 0.5 ng/mL EGF or EGF + indicated IgGs (20 µg/mL). Cell growth was assayed using MTS assays. Values shown are mean (SD). n.s.: not significant vs. control. (C) DLD-1 cells were treated for 36 h with or without 0.5 ng/mL EGF or EGF + indicated IgGs (20 µg/mL). Cell growth was assayed using MTS assays. Values shown are mean (SD). n.s.: not significant vs. control.

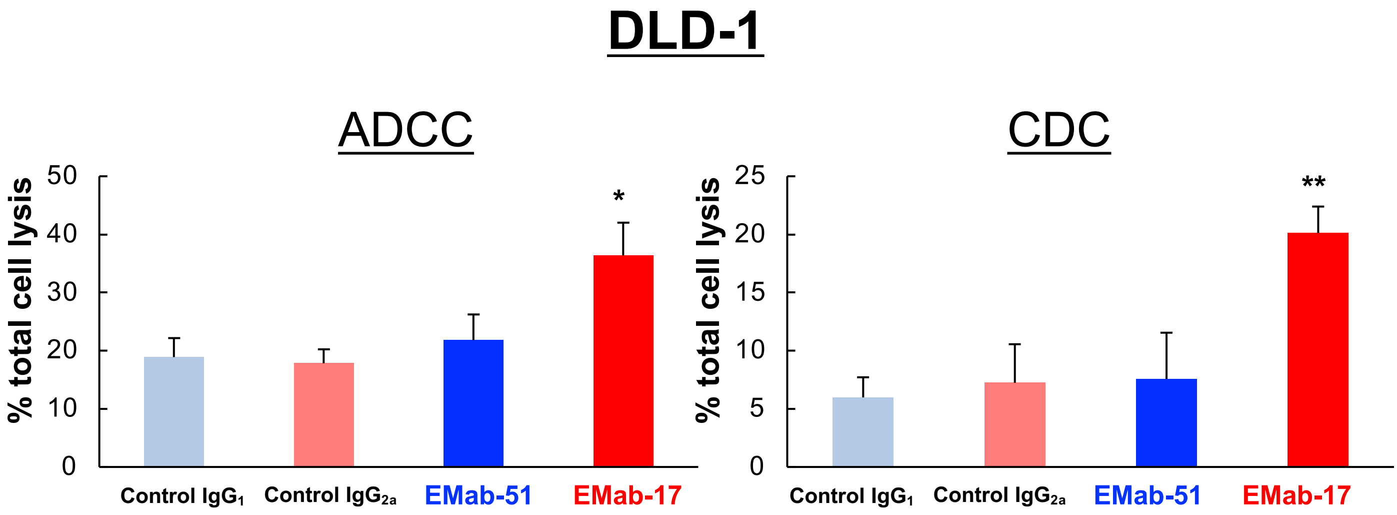

Figure S5 Determination of ADCC and CDC activities of EMab-17 or EMab-51. The ADCC and CDC activities of EMab-17 or EMab-51 against DLD-1 cells were evaluated. ADCC activity was evaluated through the calcein-AM release assay in the presence of indicated antibodies (100 µg/mL; effector/target ratio, 50). CDC activity was determined using the MTS assay in the presence of indicated antibodies (100 µg/mL) with 10% rabbit complement. The values are means (SD). *: $P < 0.05$, **: $P < 0.01$ vs. IgG_{2a}-treated control.

Ohishi T. et al. Figure S5

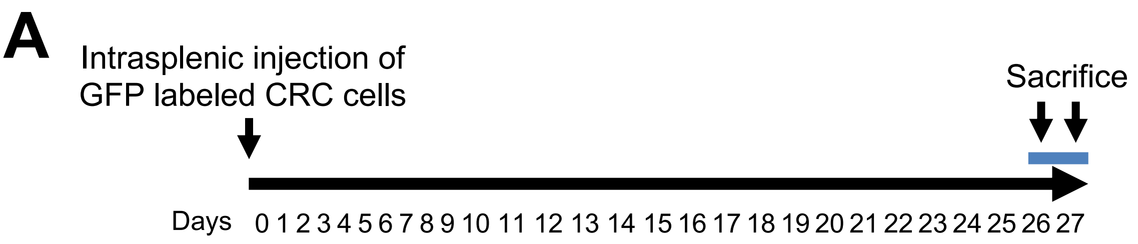

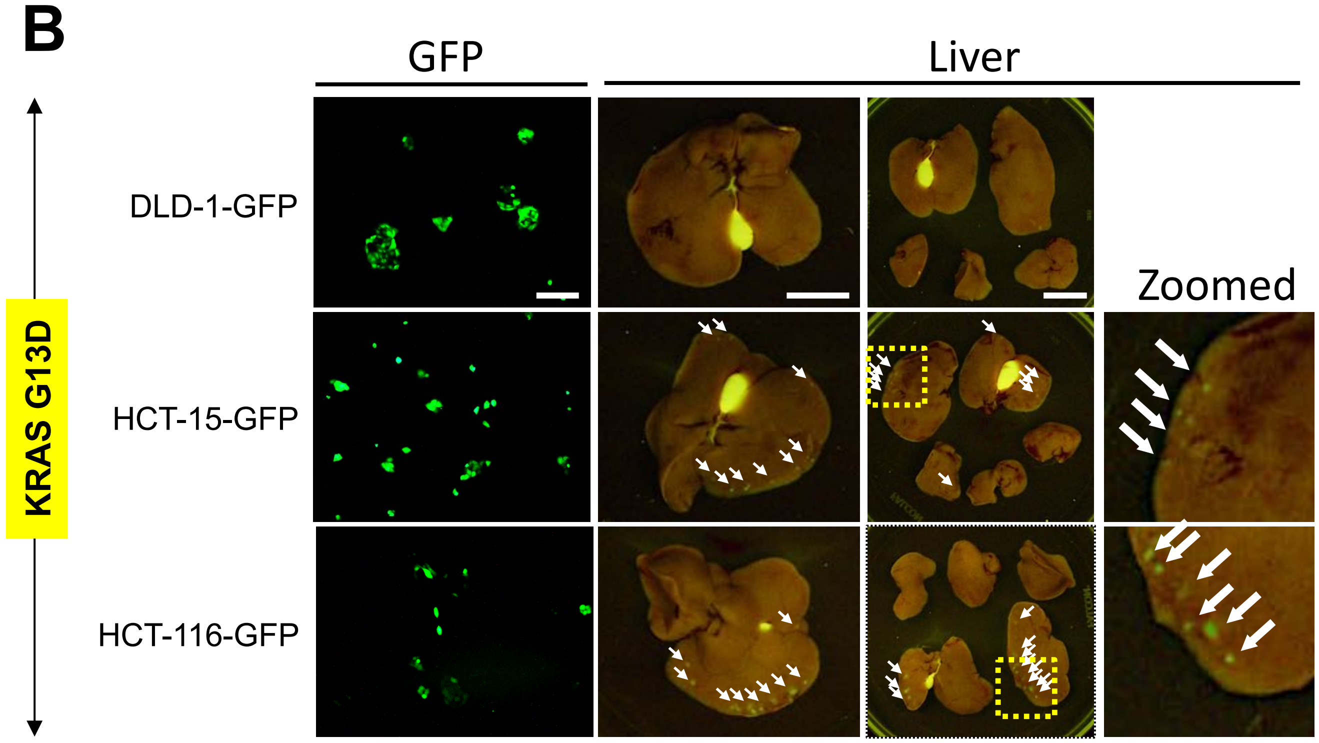

C

KRAS G13D

2.5 × 10^5 cells / mouse	DLD-1 -GFP	HCT-15 -GFP	HCT-116 -GFP
Liver metastasis	0/3 (0%)	3/3 (100%)	3/3 (100%)
Tumor growth in spleen	0/3 (0%)	3/3 (100%)	3/3 (100%)

Figure S6 Characterization of a nude mice model of CRC metastasis to the liver. (A) The experimental schedule. DLD-1-GFP, HCT-15-GFP, and HCT-116-GFP cells (2.5 × 10^5 cells suspended with Matrigel/10 μL) were injected into the spleens. Mice were sacrificed 26 or 27 days after the injection of CRC cells, and the livers were removed to determine the frequency of liver metastasis. (B) Representative images of GFP-labeled cells and the livers. The arrows point to the site of liver metastasis. Scale bar: 10 mm. The rightmost image, representing liver metastasis, shows a magnified image of the yellow dotted box in the middle image. (C) The status of CRC metastasis to the liver and tumor growth in the spleen is shown in the table. The frequency of metastasis to the liver and tumor growth in the spleen are shown in brackets.

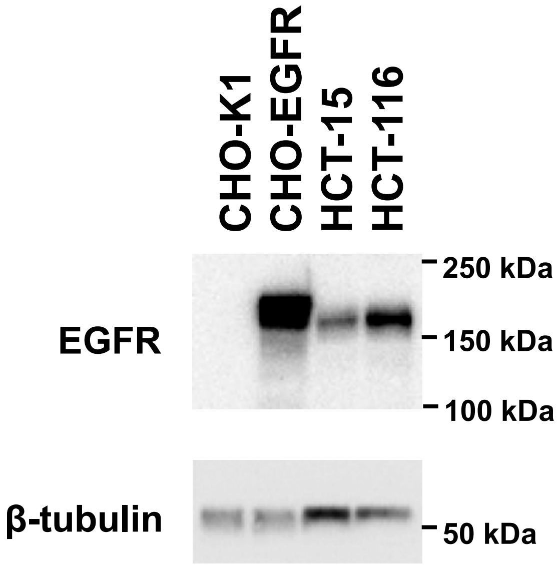

Figure S7 Western blot analysis using anti-EGFR antibody. CHO-K1, CHO/EGFR, HCT-15, and HCT-116 cells were subjected to gel electrophoresis and western blotting analysis with an anti-EGFR (CST#4267) or anti-b-tubulin antibody (CST#5346). The cell extracts from CHO-K1 and CHO/EGFR cells (10 μg of proteins) or HCT-15 and HCT-116 cells (40 μg of proteins) were loaded for each lane.

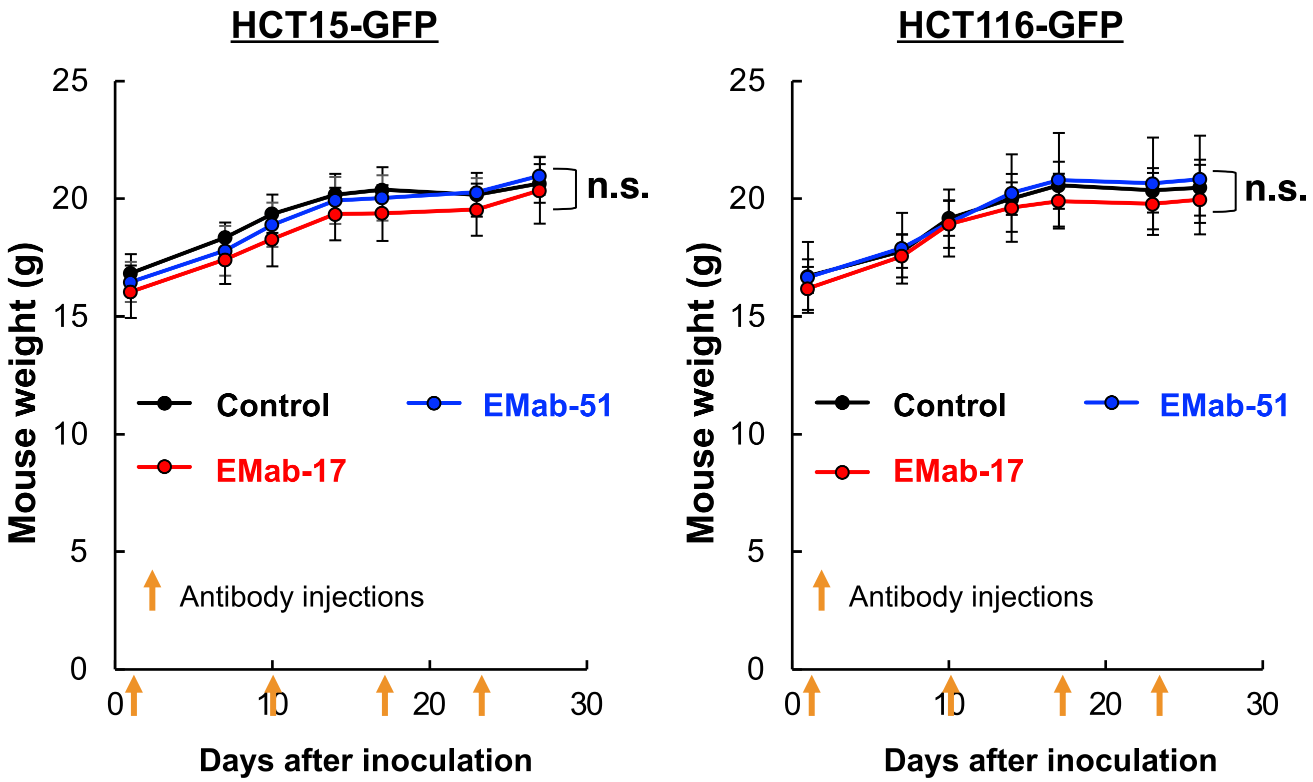

Figure S8 Body weight was not changed in mice treated with control IgG, EMab-51, and EMab-17. Change in body weight was observed in the mice from Fig. 6C. n.s.: not significant vs. control.

Ohishi T. et al. Figure S8

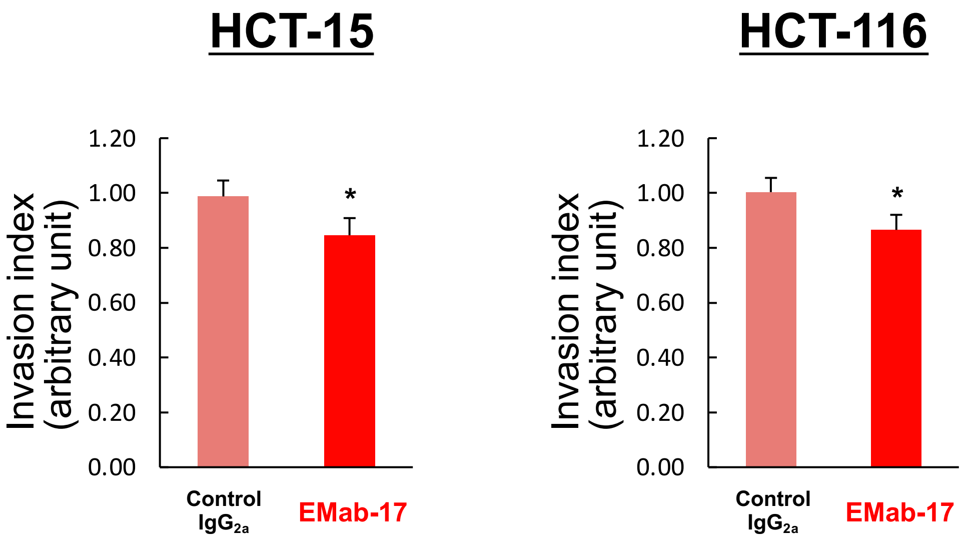

Figure S9 HCT-15 or HCT-116 cells were placed in 6-well plates 5 × 10^5 cells/well in DMEM supplemented with 10% FBS. Subsequently, the cells were incubated with the anti-EGFR antibody (EMab-17, 100 μg/mL) or control IgG_{2a} (100 μg/mL) and 10% of rabbit complement for 2 h and then analyzed by invasion assay. After 24 h incubation, invaded cells were quantitated. The values are means (SD). *: $P < 0.05$ vs. IgG_{2a}-treated control.
